# Health Care Utilisation and Transitions between Health Care Settings in the Last 6 Months of Life in Switzerland

**DOI:** 10.1371/journal.pone.0160932

**Published:** 2016-09-06

**Authors:** Caroline Bähler, Andri Signorell, Oliver Reich

**Affiliations:** Department of Health Sciences, Helsana Insurance Group, P.O. Box, 8081 Zürich, Switzerland; Mayo Clinic, UNITED STATES

## Abstract

**Background:**

Many efforts are undertaken in Switzerland to enable older and/or chronically ill patients to stay home longer at the end-of-life. One of the consequences might be an increased need for hospitalisations at the end-of-life, which goes along with burdensome transitions for patients and higher health care costs for the society.

**Aim:**

We aimed to examine the health care utilisation in the last six months of life, including transitions between health care settings, in a Swiss adult population.

**Methods:**

The study population consisted of 11'310 decedents of 2014 who were insured at the Helsana Group, the leading health insurance in Switzerland. Descriptive statistics were used to analyse the health care utilisation by age group, taking into account individual and regional factors. Zero-inflated Poisson regression model was used to predict the number of transitions.

**Results:**

Mean age was 78.1 in men and 83.8 in women. In the last six months of life, 94.7% of the decedents had at least one consultation; 61.6% were hospitalised at least once, with a mean length of stay of 28.3 days; and nursing home stays were seen in 47.4% of the decedents. Over the same time period, 64.5% were transferred at least once, and 12.9% experienced at least one burdensome transition. Main predictors for transitions were age, sex and chronic conditions. A high density of home care nurses was associated with a decrease, whereas a high density of ambulatory care physicians was associated with an increase in the number of transitions.

**Conclusions:**

Health care utilisation was high in the last six months of life and a considerable number of decedents were being transferred. Advance care planning might prevent patients from numerous and particularly from burdensome transitions.

## Introduction

As the end-of-life is likely to be linked to chronic illnesses, deeper knowledge about the coordination of care at the end-of-life is of great importance [1]. With increasing life expectancy concerns about higher health care utilisations are rising. This is especially true for a country like Switzerland with one of the highest life expectancy in women, and the highest life expectancy in men worldwide, according to the Organisation for Economic Co-operation and Development (OECD) [2]. Many efforts are undertaken in Switzerland to enable older and/or chronically ill patients to stay home longer. However, as shown in previous foreign studies, one of the consequences might be an increased need for hospitalisations at the end-of-life [3,4]. On the one hand, hospitalisations might turn out to be necessary when patients become more dependent, as dying at home usually requires formal as well as informal care; dying in a hospital was almost four times more likely for those who only received informal care, in contrast to those who received combined formal and informal care [5]. Likewise, having an infection was associated with terminal hospitalisation, whereby home-to-hospital was found to be the most frequent trajectory in the Netherlands [6]. According to van den Block and colleagues [3], however, final transition to hospital was frequently due to curative or life-prolonging reasons.

On the other hand, the presence of professional caregivers in nursing homes allow for better care at the end-of-life, which might be another reason for the fewer number of hospital admissions in nursing home residents [7]. In line with this, several studies have found a lower rate of health care utilisation and transitions for the oldest old at the end-of-life compared to younger decedents due to the higher proportion of nursing home residents in the highest age group [8,9]. In Switzerland, data on age differences in the end-of-life treatment are scarce.

Hospitalisations at the end-of-life go along with burdensome transitions for patients [10] and higher health care costs for the society. Moreover, transitions at the end-of-life can be very distressing. They are subject to discontinuity of care, as they always go with a loss of information between patient and caregiver, and may lead to decreased quality of care at the end-of-life [10,11]. Thus, it is believed that especially multiple changes in settings and care providers during the end-of-life may attenuate the quality of life [6,12]. However, the evidence regarding quality of life at the end-of-life is weak [13]. Finally, transitions between health care settings have been proposed as indicators for quality of end-of-life care [14].

However, previous research on end-of-life transitions has mainly focused on specific groups of decedents such as elderly individuals or cancer patients [5,15], or specific settings [7,16]. Research combining health care utilisation and transitions between health care settings in the last six months of life and its associated factors have not been studied often in Europe, and—to the best of our knowledge—not at all in Switzerland.

### Objectives

The present study focuses on the care delivering processes in health care in Swiss individuals aged 18 years and older. We aimed to examine the health care utilisation (consultations, hospitalisations as well as nursing home admissions), the number of transitions between health care settings in the last six months of life and the proportion of decedents with burdensome transitions (as defined by Teno et al. [10]), taking into account individual and regional factors. In particular, we analysed possible age differences in health care utilisation incurred by decedents.

We hypothesise that the rates of health care utilisation are lower in the oldest age groups compared with younger decedents. Furthermore, we assume that transitions are common in the last six months of life, resulting in half of the decedents with at least one transition at the end-of-life, whereby transitions are more common among community-dwelling individuals compared with nursing home residents.

Knowledge in the field of health care utilisation and transitions is important for health care providers and policy-makers as well as patients when debating on the future directions of the health care system, especially in terms of planning and resource allocation.

## Data and Methods

### Study design

Analyses conducted for the purpose of this study are based on claims data of decedents, who were insured at the Helsana Group, the leading health insurer in Switzerland. The Helsana database included mandatory health insurance claims from approximately 1.2 million persons, covering about 15% of the whole Swiss population, including individuals from all ages and from all 26 cantons.

Since all data were anonymised, retrospective, pre-existing, and de-identified to protect the privacy of patients, physicians, and hospitals, this study was exempted from ethics committee approval as per the Swiss Federal Law on data protection. The study protocol was approved by the Helsana Group.

### Study Population

The study population consists of 11'310 insurants aged 18 years and older who died in 2014, and of whom data on the last year of life were available. 39 (0.3%) decedents had to be excluded due to missing data (e.g. individuals who live abroad). In 293 (2.6%) nursing home residents no detailed information on the type of medication was available because medical costs were covered by a fixed rate. But, as exclusion of these patients did hardly alter the results, these decedents were also included in the analyses.

### Measures

Health care utilisation included consultations, hospitalisations, nursing home admissions and transitions between health care settings in the last six months of life. The number of consultations was divided by primary care physicians, specialists and hospital outpatient visits and comprised face-to-face consultations, phone consultations and home visits. Besides, the number of hospitalisations and the number of nursing home admissions, as well as the median length of stay, if any, was calculated. Further, the place of death (hospital, nursing home, and home/others) was assessed by means of the last claim received. The claims contain the date and the duration of the specific treatment which allow us to compare it with the date of death. If there are several claims covering the date of death, hospital is taken as place of death. A transition was defined as a change in the health care setting as identified by the claims data. Transition trajectories (pathways) were defined as combinations of changes in these health care settings [6,10,17]. Burdensome transitions were defined as 3 or more hospitalisations in the last 90 days of life, or at least one transition in the last 3 days of life [10].

The total costs covered by the compulsory health insurance were calculated on the patient-level, including inpatient, outpatient and medication costs, as well as costs concerning laboratory tests and medical devices.

Individual factors included age group (18–64, 65–74, 75–84, 85–94, 95 and over), sex, health insurance plan (being in a managed care model, having supplementary insurance (used as a proxy for socioeconomic status) and the type of residence (urban vs. rural [18])). The age distribution of the decedents in the lowest group is as follows: 192 (16.3%) of the decedents were aged 18 to 44 years, 303 (25.7%) were aged 45 to 54 years, and 685 (58.1%) of the decedents were aged 55 to 64 years. Chronic conditions were identified using the Pharmacy-based Cost Group (PCG) model by Huber et al. [19], which is based on the Anatomical Therapeutic Chemical (ATC) classification system and which differentiates between 22 different conditions: Acid related disorders, Bone diseases (osteoporosis), Cancer, Cardiovascular diseases (incl. hypertension), Dementia, Diabetes mellitus, Epilepsy, Glaucoma, Gout/Hyperuricemia, HIV, Hyperlipidemia, Intestinal inflammatory diseases, Iron deficiency anemia, Migraines, Pain, Parkinson's disease, Psychological disorders (sleep disorder, depression), Psychoses, Respiratory illness (asthma, COPD), Rheumatologic conditions, Thyroid disorders, and Tuberculosis. Chronic conditions were categorised into three groups (0–1, 2–4, and 5 or more) to account for disease-dependent differences on the number of transitions. Unfortunately, the cause of death was not available for the present analysis. However, most people suffer several years from one or more chronic diseases before they die, and less than 5% of the people die suddenly i.e. due to a fatal accident or a myocardial infarction in Switzerland [20].

Regional variables included the density of nursing home beds per 1000 inhabitants aged 65 years and older derived from the statistics of socio-medical institutions [21], the number of home care nurses per 1000 inhabitants derived from the statistics of home care and nursing services [22], and the number of ambulatory care physicians per 100'000 inhabitants, which was derived from the statistics of the Swiss Medical Association [23]. The density of medical supply was each assessed on the cantonal level and dichotomised into cantons with scores above and below the median score, respectively. However, as the density of nursing home beds did not show a significant effect in any of the regression models, this variable was excluded. Finally, the Hospital Service Areas (HSA), which was constructed by analysing the discharge data of all acute hospitals in Switzerland 2008–2010, was included in the regression models [24–26].

### Statistical analysis

Descriptive statistics were used to analyse the health care utilisation by age group, including consultations, hospitalisations, nursing home admissions and transitions between health care settings in the last six months of life, as well as the site of death. Figures are provided of the number of transitions by age group and sex using boxplots. In the presence of a skewed distribution and due to the excessive number of zeros, a Zero-Inflated Poisson Generalized Linear Model was used to predict the number of transitions by way of the following two equations:

Eq 1 depicts the binomial model used,

$$\begin{array}{ll} \text{log}\langle \frac{P\langle Z_{i}=1\rangle }{1-P\langle Z_{i}=1\rangle }\rangle & =\beta_{0}+\beta_{1,k}\cdot AGE_{i,k}+\beta_{2}\cdot SEX_{i}+\beta_{3,k}\cdot AGE_{i,k}:SEX_{i}+\beta_{4,l}\cdot CHRON_{i,l}+\beta_{5}\cdot HDAMB_{i}\\ & \;+\beta_{6}\cdot HDNRS_{i}+\beta_{7,m}\cdot HSA_{i,m}+\beta_{8,n}\cdot AREA_{i,n}+\beta_{9}\cdot MC_{i}+\beta_{10}\cdot PRIV_{i} \end{array}$$ (1)

whereas Eq 2 describes the Poisson-model (assuming the same auxiliary information):

$$\begin{array}{ll} \text{log}\langle \mu_{i}\rangle & =\beta_{0}+\beta_{1,k}\cdot AGE_{i,k}+\beta_{2}\cdot SEX_{i}+\beta_{3,k}\cdot AGE_{i,k}:SEX_{i}+\beta_{4,l}\cdot CHRON_{i,l}+\beta_{5}\cdot HDAMB_{i}\\ & \;+\beta_{6}\cdot HDNRS_{i}+\beta_{7,m}\cdot HSA_{i,m}+\beta_{8,n}\cdot AREA_{i,n}+\beta_{9}\cdot MC_{i}+\beta_{10}\cdot PRIV_{i} \end{array}$$ (2)

The used response and explanatory variables were:

Y - Number of transitions in the last 6 months of life

AGE - Age at time in (k+1) = five groups

SEX - Sex of patient: dummy variable equal to 1 if decedent was female and zero if male

CHRON - Number of chronic conditions, in (l+1) = three groups: 0–1, 2–4 and 5+

HDAMB - High density of ambulatory care physicians in the canton

HDNRS - High density of home care nurses

HSA - Hospital service area (m) according to the domicile of the insured

AREA - Region according to the domicile of the insured in (n+1) = two categories: CITY (reference), RURAL.

MC - Managed care health plan type: dummy variable equal to 1 if member chose a managed care health plan and zero otherwise

PRIV - Supplementary private hospital insurance: dummy variable equal to 1 if decedent possessed additional private hospital insurance coverage and zero otherwise

Incidence risk ratios (IRRs) are shown for the number of transitions and odds ratios (OR) for the dichotomous outcomes [27]. Individual and regional variables were included as independent factors. We modelled the sex-related difference in the age-dependency of the utilisation and costs with interaction terms. The influence of each age group on the number of transitions is shown separately for men and women, taking into account the effect of the interaction term. We did not perform negative binomial modelling due to the lack of overdispersion.

Associations between the total health care costs and the number of transitions were assessed by means of linear regression, taking into account further patient-level and regional-level variations in health care. Again, the influence per age group is shown separately for men and women, thereby including the effect of the interaction term. Log-transformation was applied to account for the non-linear relationship between predictors and response variable. Model quality was quantified using R^2^. All analyses were conducted using R statistics, version 3.2.0.

## Results

**Fig 1:**
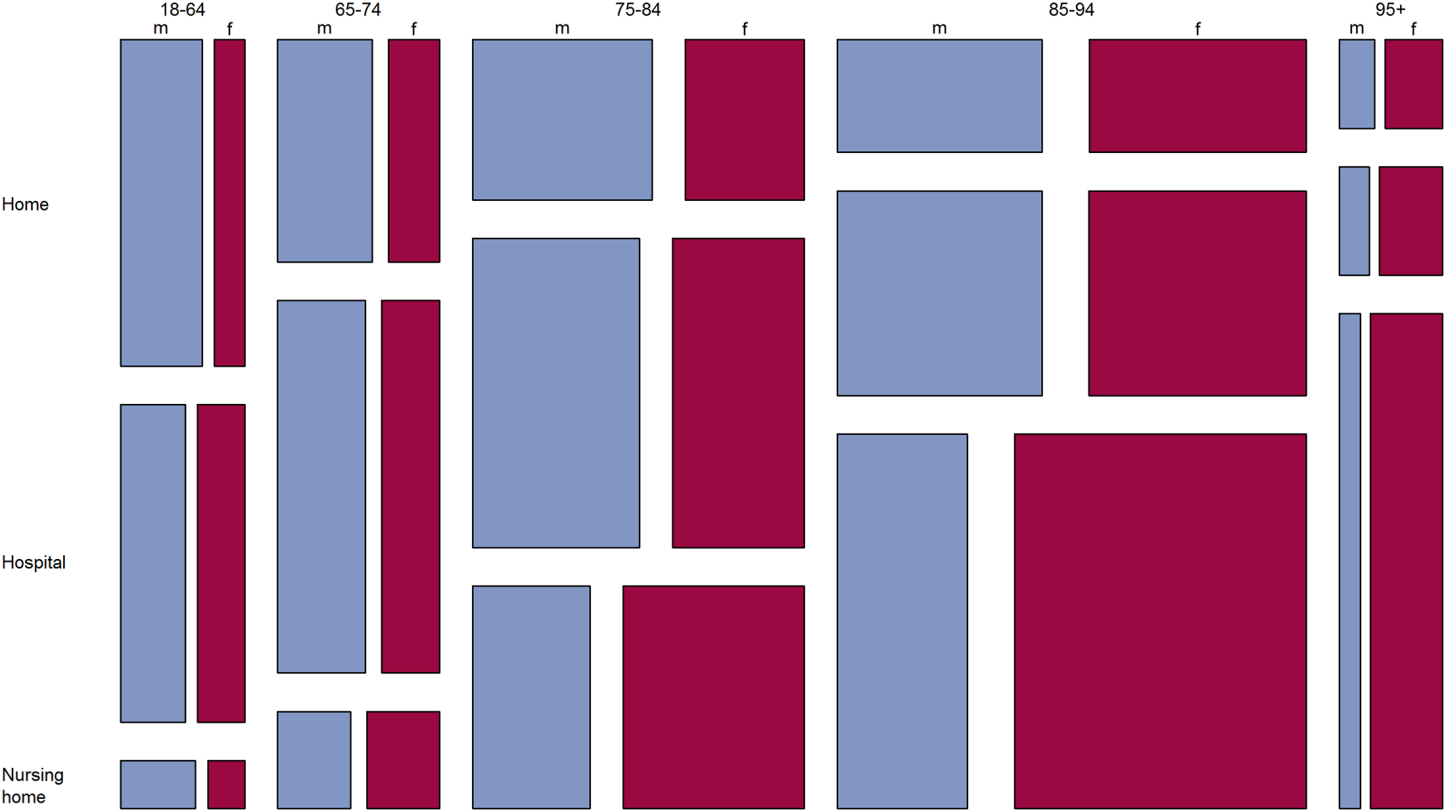
**Mosaic plot of place of death as a function of age group and sex** m = male, f = female, n = 11310.

Of the 11'310 decedents, 5989 (53.0%) were female. Mean (± SD) age was 78.1 (± 13.4) in men and 83.8 (± 11.6) in women. Six months before death, 7156 (63.3%) of the study population resided at home, 490 (4.3%) were hospitalised, and 3664 (32.4%) lived in a nursing home. The mean (± SD) number of chronic conditions amounted to 4.0 (± 2.3) in men and 4.3 (± 2.2) in women. Characteristics of the decedents are shown in Table 1. While being in a managed care model decreased continuously with increasing age (39.2% in the youngest vs. 23.2% in the oldest age group, p<0.001), having a supplementary hospital insurance increased slightly with increasing age (13.5% in the youngest vs. 16.5% in the oldest age group, p<0.001). Almost 40% of the individuals died in nursing homes in 2014, and only a little more than one fifth died outside a health care institution (Fig 1).

**Table 1:** Characteristics of decedents in 2014 (n = 11'310).

	N	%
Female sex	5989	53.0
Age (years)		
18–64	1180	10.4
65–74	1539	13.6
75–84	3153	27.9
85–94	4458	39.4
95+	980	8.7
Chronic conditions		
0–1	1479	13.8
2–4	4549	42.3
5+	4723	43.9
Place of death		
Hospital	4249	37.6
Nursing home	4425	39.1
Home	2636	23.3
Type of residence (rural area)	7620	67.4
Managed care model	3472	30.7
Supplementary hospital insurance	2191	19.4

The health care utilisation of the decedents is shown in Table 2. The total number of consultations was on average 14.8 (± 12.9) per patient in the last six months of life in those 94.7% patients with at least one consultation. Of those decedents, 89.6% were seen by more than one primary care physician in the last six months of life, and 51.4% consulted more than one specialist over the same time period. Of the total study sample, 61.6% (6972) have been hospitalised at least once. Their mean (± SD) length of stay was 28.3 (± 30.4) days. Nursing home stays were seen in 47.4% (5363) of the decedents with a mean (± SD) of 136.5 (± 63.2) days in those nursing home residents. The percentage of individuals with at least one hospitalisation and with at least one transition decreased with increasing age (except for the youngest age group), whereas, unsurprisingly, the percentage of individuals with a nursing home admission increased with increasing age.

**Table 2:** Health care utilisation of decedents in their last 6 months of life (n = 11'310).

Age groups					
n (%)	18–64 (n = 1180)	65–74 (n = 1539)	75–84 (n = 3153)	85–94 (n = 4458)	95+ (n = 980)
Individuals with hospitalisation(s)	732 (62.0)	1142 (74.2)	2226 (70.6)	2531 (56.8)	341 (34.8)
Length of stay^1^ (median, IQR)	22 (31)	22 (32)	19 (26)	15 (22)	11 (16)
Individuals with nursing home admission(s)	112 (9.5)	311 (20.2)	1275 (40.4)	2888 (64.8)	777 (79.3)
Length of stay^1^ (median, IQR)	86 (151)	121 (152)	166 (129)	178 (69)	181 (10)
Individuals with at least one transition	760 (64.4)	1162 (75.5)	2307 (73.2)	2671 (59.9)	390 (39.8)
Individuals with ≥ 3 hospitalisations in last 90 days^1^	85 (11.2)	125 (10.8)	127 (5.5)	66 (2.5)	5 (1.3)
Individuals with ≥ 1 transition in last 3 days^1^	136 (17.8)	192 (16.5)	365 (15.8)	404 (15.1)	58 (14.9)
Individuals with consultation(s)	1054 (89.3)	1453 (94.4)	3038 (96.4)	4242 (95.2)	926 (94.5)
Number of consultations^1^ (mean, SD)	19.3 (16.9)	18.9 (15.1)	15.9 (13.7)	12.6 (10.0)	10.0 (7.3)
by primary care physicians	5.1 (6.5)	6.4 (6.6)	7.7 (7.0)	8.4 (7.3)	7.7 (6.4)
by specialists	4.5 (8.0)	3.6 (6.7)	2.8 (5.6)	2.0 (4.4)	1.4 (3.7)
hospital outpatient visits	9.7 (13.4)	8.9 (12.6)	5.4 (10.6)	2.1 (5.9)	0.9 (2.5)

^1^ in decedents with at least one admission, transition or consultation, respectively

**Fig 2:**
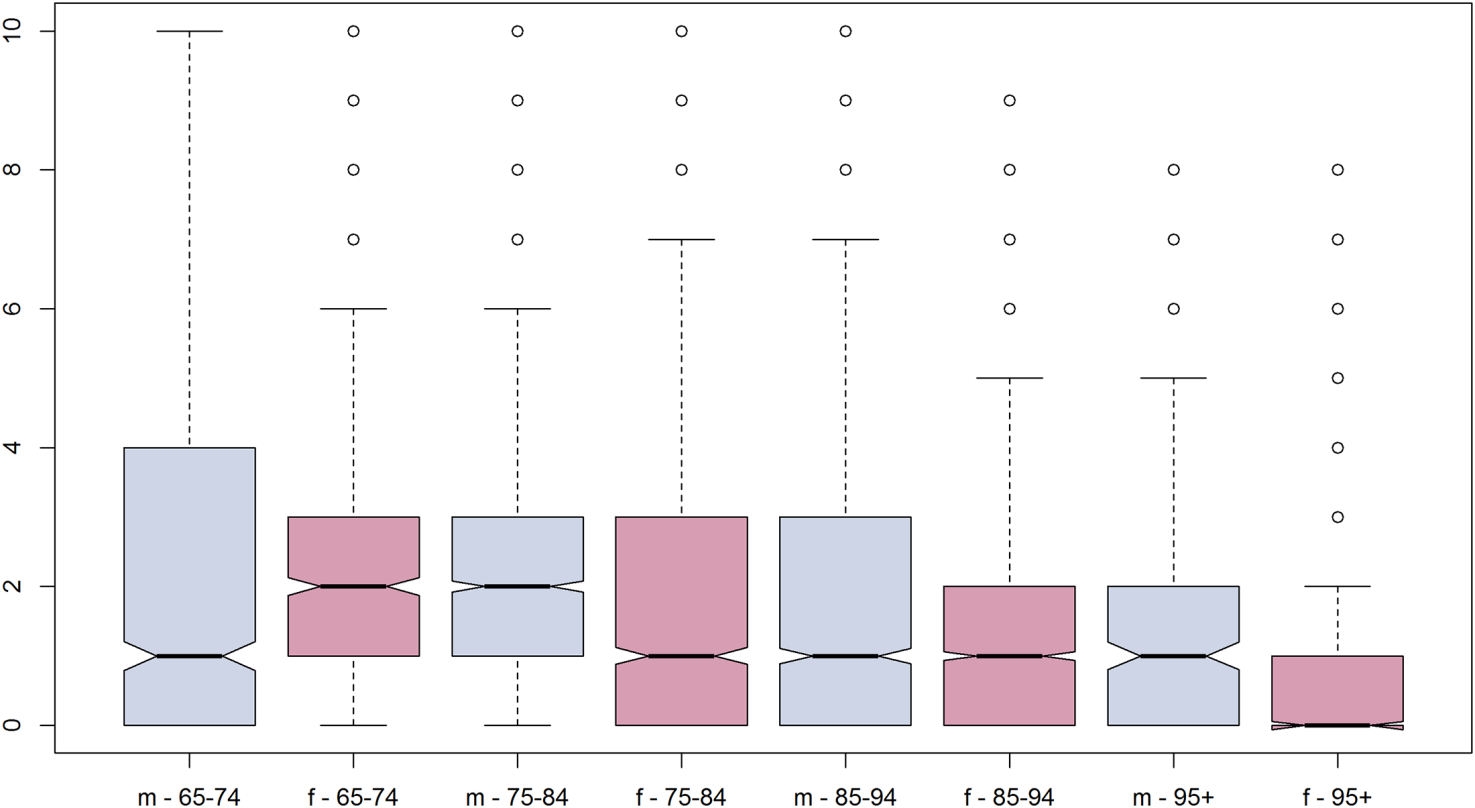
Boxplot on the number of transitions in a Swiss elderly population, divided by sex and age group (n = 11310).

Overall, 64.5% (7290) of the decedents were transferred at least once in the last six months of life. The mean (± SD) number of transitions was 2.8 (± 2.0; range 1–21) in those who had been transferred at least once. The number of transitions significantly decreased with increasing age in the elderly (besides in the lowest age category), and it was significantly lower in women compared to men in the last six months of life (Fig 2). In total, 12.9% (1462) experienced at least one burdensome transition; 9.3% (1054) of the decedents had at least one transfer in the last 3 days of life, 2.7% (307) had 3 or more hospitalisations in the last 90 days of life, and 0.9% (101) had both. Most frequently, patients were transferred from home to hospital. This pathway accounted for 50.0% of all transfers, while transfers from nursing home to hospital only made up 14.2%. Of the 4020 decedents who have not had any transition, 1541 (38.3%) stayed at home, 2426 (60.3%) stayed in a nursing home and 53 (1.3%) patients were in hospital during the last six month of life.

Being female was associated with lower odds of being transferred, whereas having supplementary hospital insurance and having two or more chronic conditions increased the chances of being transferred at least once (Table 3a). Men aged 75 to 84 years had the highest odds of being transferred compared to men aged 18 to 64 years (OR = 2.59 [2.01–3.33], p<0.001). In women, the odds of being transferred decreased with increasing age. Interestingly, neither stationary (HSA) nor ambulatory medical supply (density of ambulatory care physicians, density of home care nurses), nor the type of residence or being in a managed care model were associated with transition occurrence (Table 3).

**Table 3:** Binomial model on transition occurrence as part of the zero-inflated Poisson regression model on the number of transitions in the last 6 months of life (n = 10751). Odds ratios for HSA are not listed in the model.

	OR^1^ (95% CI)	p-value^2^
Age group by male sex		
18–64 (male)	1.000	
65–74 (male)	2.061 (1.575–2.698)	***
75–84 (male)	2.589 (2.014–3.328)	***
85–94 (male)	1.822 (1.436–2.311)	***
95+ (male)	0.837 (0.568–1.232)	
Age group by female sex		
18–64 (female)	1.000	
65–74 (female)	0.937 (0.593–1.480)	
75–84 (female)	0.628 (0.423–0.934)	*
85–94 (female)	0.300 (0.206–0.435)	***
95+ (female)	0.147 (0.097–0.221)	***
Number of chronic conditions		
0–1	1.000	
2–4	1.870 (1.533–2.282)	***
5+	2.562 (2.102–3.122)	***
High density of ambulatory care physicians	0.486 (0.197–1.199)	
High density of home care nurses	2.284 (0.722–7.219)	
Type of residence (rural area)	0.937 (0.823–1.067)	
Managed care	1.081 (0.954–1.226)	
Supplementary hospital insurance	1.248 (1.077–1.447)	**

^1^ OR = odds ratio

^2^ *: p<0.05; ** p<0.01; ***: p<0.001

**Fig 3:**
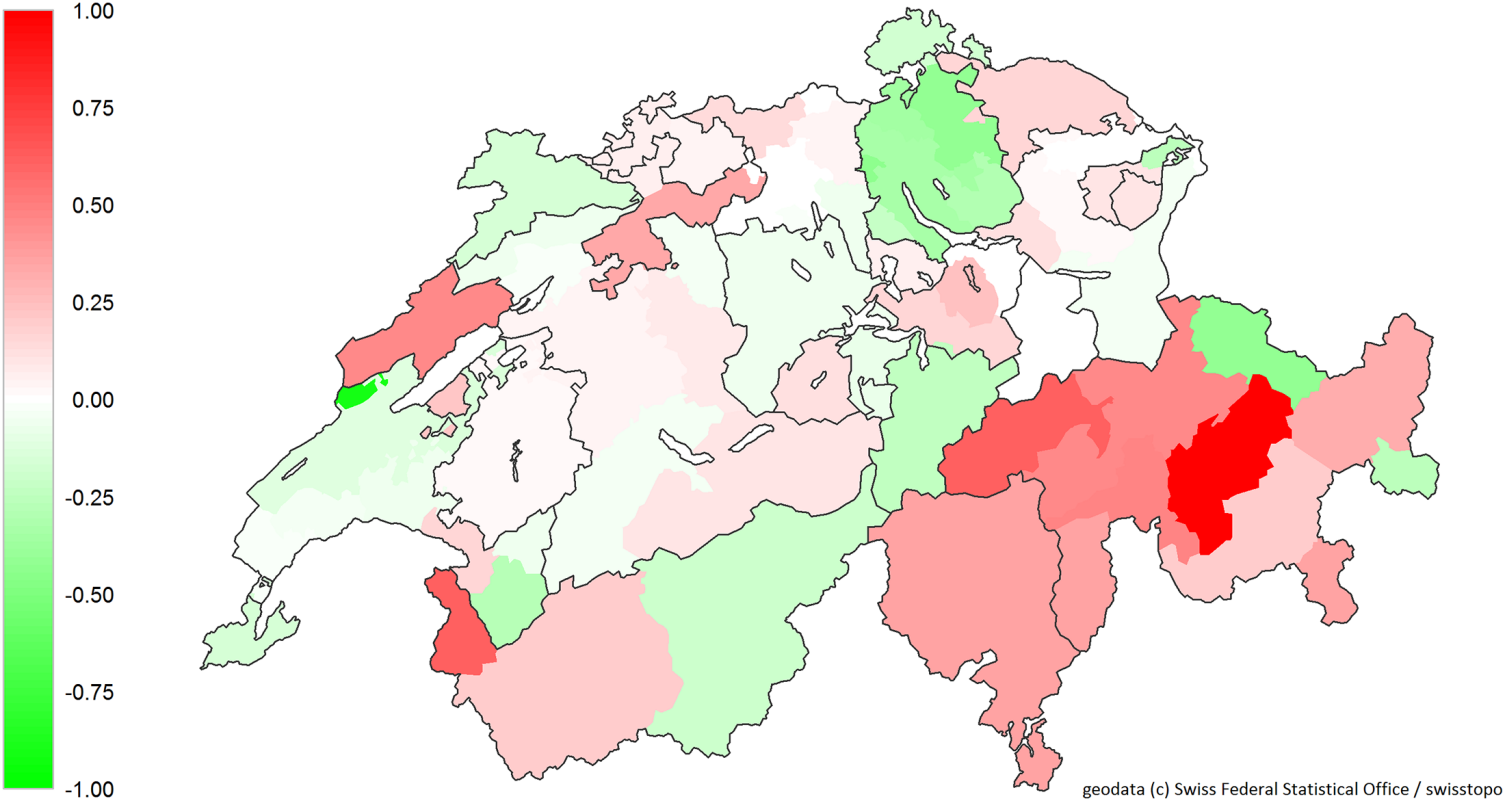
The specific additive component of the Hospital Service Areas to the estimator for transitions, adjusted for individual and further regional factors.

The number of transitions decreased with increasing age in both men and women, whereby the decrease was steeper in men. The number decreased by 37.6% in men aged 95 years and older and by 33.0% in women of the same age group, compared with their counterparts in the youngest age group (Table 4). The number of transitions was 1.7-fold and 2.2-fold in patients with 2–4 and in patients with 5+ chronic conditions, respectively. A high density of home care nurses was associated with a decrease in the number of transitions by 31.7%, whereas a high density of ambulatory care physicians increased the number of transitions by 51.1% (Table 4); however, these percentages must be interpreted with caution, as the confidence intervals are relatively wide (IRR = 0.68 [0.51–0.91], p<0.01, and IRR = 1.51 [1.22–1.88], p<0.001, respectively). The type of residence and being in a managed care model only showed marginal effects. Regarding regional variations, the French speaking part showed lower number of transitions compared to the German and Italian speaking part of Switzerland. Regional (HSA) variations on the number of transitions are shown in Fig 3.

**Table 4:** Poisson regression model on the number of transitions as part of the zero-inflated Poisson regression model on the number of transitions in the last 6 months of life (n = 10751). Odds ratios for HSA are not listed in the model.

	IRR^1^ (95% CI)	p-value^2^
Age group by male sex		
18–64 (male)	1.000	
65–74 (male)	0.839 (0.782–0.901)	***
75–84 (male)	0.745 (0.698–0.795)	***
85–94 (male)	0.660 (0.616–0.707)	***
95+ (male)	0.624 (0.537–0.725)	***
Age group by female sex		
18–64 (female)	1.000	
65–74 (female)	0.964 (0.881–1.056)	
75–84 (female)	0.793 (0.730–0.861)	***
85–94 (female)	0.748 (0.690–0.811)	***
95+ (female)	0.670 (0.590–0.761)	***
Number of chronic conditions		
0–1	1.000	
2–4	1.689 (1.555–1.834)	***
5+	2.280 (2.103–2.471)	***
High density of ambulatory care physicians	1.511 (1.216–1.877)	***
High density of home care nurses	0.683 (0.514–0.907)	**
Type of residence (rural area)	1.040 (1.003–1.080)	*
Managed care	1.053 (1.017–1.089)	**
Supplementary hospital insurance	1.082 (1.041–1.124)	***

^1^ IRR = incident risk ratio

^2^ *: p<0.05; ** p<0.01; ***: p<0.001

As far as burdensome transitions were concerned, the main predictors remained age, sex and chronic conditions, and, to a lesser extent, the HSA: the odds of having experienced a burdensome transition decreased with increasing age: OR = 1.06 (0.86–1.29, p = 0.549) in decedents aged 65–74, OR = 0.81 (0.68–0.98, p<0.05) in decedents aged 75–84, OR = 0.56 (0.46–0.67, p<0.001) in decedents aged 85–94, and OR = 0.33 (0.24–0.45, p<0.001) in those aged 95 and older, respectively, compared to men and women aged 18 to 64 years. Women had a decreased odds of 0.87 (CI: 0.78–0.98, p<0.05) of being transferred in a burdensome manner. Again, a higher number of chronic conditions was associated with a higher odds of having experienced a burdensome transition. When looking at the linguistic regions, decedents in the Italian speaking part had a 1.67 (CI: 1.33–2.08, p<0.001) higher odds, whereas the French speaking part had a 0.84 (CI: 0.71–0.98, p<0.05) lower odds of having experienced at least one burdensome transition compared to the German speaking part. All other variables showed no significant relation to the occurrence of any burdensome transition.

Table 5 shows the linear regression model on the total health care costs in the last six months of life. Each additional transition was associated with a 8.3% increase in costs, after adjustment for a variety of individual and regional variables like hospital admission or nursing dependency. Costs were highest in those aged 65 to 74 years for both genders. Total costs more than doubled for decedents dying in a hospital or nursing home. A high density of home care nurses was associated with a decrease in total costs of 31.1%, whereas a high density of ambulatory care physicians was related to an increase of 25.0% of the total health care costs. Again, due to the wide confidence intervals these percentages must be interpreted with caution.

**Table 5:** Multiple linear regression model on the total health care costs in the last 6 months of life (n = 10659).

	β (95% CI)	p-value^1^
Age group by male sex		
65–74 (male)	1.000	
18–64 (male)	0.772 (0.717–0.830)	***
75–84 (male)	0.877 (0.824–0.932)	***
85–94 (male)	0.751 (0.705–0.800)	***
95+ (male)	0.789 (0.706–0.882)	***
Age group by female sex		
65–74 (female)	1.000	
18–64 (female)	1.098 (0.998–1.209)	
75–84 (female)	0.873 (0.812–0.939)	***
85–94 (female)	0.849 (0.791–0.910)	***
95+ (female)	0.944 (0.865–1.030)	
Number of transitions	1.083 (1.073–1.093)	***
Number of chronic conditions		
None	1.000	
1	1.677 (1.601–1.757)	***
2+	1.887 (1.796–1.982)	***
Site of death		
Home	1.000	
Hospital	2.196 (2.100–2.297)	***
Nursing home	2.330 (2.185–2.484)	***
Number of consultations	1.022 (1.021–1.023)	***
Hospital admission	1.548 (1.478–1.621)	***
Nursing home admission	1.282 (1.210–1.359)	***
Nursing dependency	1.261 (1.218–1.306)	***
Managed care	0.947 (0.917–0.977)	***
High density of ambulatory care physicians	1.250 (1.029–1.519)	*
High density of home care nurses	0.689 (0.525–0.905)	**
R2	0.548	

^1^ *: p<0.05; ** p<0.01; ***: p<0.001

## Discussion

The present study in a Swiss adult population has shown that health care utilisation was high at the end-of-life. We found a decrease in home deaths and hospital deaths, and an increase in people dying in nursing homes when comparing our results to those of a previous Swiss study, where 38.4% of decedents died in hospitals, 35.1% died in nursing homes and 26.6% died at home [1]. The percentage of nursing home deaths is also higher compared to the outcomes in 85'129 Swiss decedents of the years 2007 and 2008, where 36.0% died in nursing homes [28]. Moreover, our percentage of home deaths is also lower compared to another study on administrative data in people aged 40 years and older in Belgium resulting in 27% home deaths [8]. This trend in home deaths is contrary to what the policy was seeking, and to what international studies [29,30] as well as a representative Swiss survey [31] have revealed: that more than half of all patients, and three fourth of the Swiss people prefer to die at home, respectively. In contrast, the reduction in in-hospital deaths is a more favourable trend. However, Pollock [32] doubts that home is always the best place to die and proposes instead that greater efforts should be made to offer the best possible support for patients dying in hospitals.

Overall, we found a lower rate of health care utilisation (including transitions) and health care costs in the older age groups for both genders. According to the present findings almost 62% were hospitalised at least once in the last six months of life. This is comparable to other research findings, where over 60% of people were hospitalised at least once in the last three months of life in Belgium [7]. In hospitalised patients, the median length of stay decreased with increasing age in our data, which is in line with another Belgian study [8]. However, patients without any hospitalisation were not excluded in their calculations. Likewise, the number of contacts with general practitioners ranged between 5 and 14 in this same time span, and increased with increasing age category in their findings, which is also in line with our findings, where the mean number of consultations by primary care physicians increased from 5 to 8 with increasing age (except for those 95 years and older). In contrast, the total number of consultations decreased with increasing age due to the fact that visits to specialists and hospital outpatient visits were more frequent in the younger age categories.

About two thirds (64.5%) experienced at least one transition in our study. And as much as 12.9% experienced one or more burdensome transition. Half of all transitions occurred from home to an acute hospital, whereas only 14.2% occurred from a nursing home to an acute hospital. Individual factors were associated with the odds of being transferred, and individual as well as regional variables were related to the number of transitions. Older age (in both men and women) and a high density of home care nurses were associated with a decreased number of transitions, whereas a high density of ambulatory care physicians was associated with an increase. The reason why a greater density of home care nurses is associated with a lower hospitalisation rate is likely due to the broader, diel and more specialised range of health care services provided in those areas, which may translate into better pain and symptom management. Costs spent on home care staff may therefore be set off by lower costs spent on hospitalisations. Furthermore, the number of transitions was lower in the French speaking regions of Switzerland, where comprehensive palliative care is more common and has a longer tradition compared to the other regions. The density of ambulatory care physicians is more pronounced in urban areas, due to higher numbers of specialists. This fact might not be fully accounted for by the inclusion of the type of residence, where rural and agglomeration areas have been combined. Therefore, we believe that the higher likelihood of additional transitions in areas with a higher density of physicians is at least partly due to urbanity. However, further research is needed. In contrast, in the logistic regression model, only individual factors were associated with the likelihood of being transferred, whereas regional variables revealed no significant association. While we found a decreasing likelihood of a transition with increasing age in women, the odds of being transferred increased in men up to the age of 75–84 years and decreased thereafter. This sex-related differences might be due to the fact that men stay home longer than women and are taken care of by their wives or relatives.

We can only speculate why regional variations did not alter the results in the logistic model. As the overall density of hospitals, nursing homes and ambulatory care physicians is rather high and the regional variations are small compared to other (mostly larger) countries, we suppose that all patients in need obtain medical attendance within a brief span. In line with our findings, the ratio of the number of hospital beds per 1000 inhabitants did not influence the hospitalisation rates in Belgium [7].

Besides a high number of transitions at the end-of-life, particularly those transitions from home to hospital, we have also found higher costs in those being transferred. Again, a high density of home care nurses was associated with decreased, whereas a high density of ambulatory care physicians was associated with increased total health care costs.

Our results are in line with previous research findings where 67% of the decedents were transferred at least once during the last three months [6]. In the SENTI-MELC Study, only 38% of the decedents had no transition in the last three months of life [33]. Our results are also consistent with a recently published international study, in which data were gathered retrospectively by general practitioners networks [3]; more than one in two patients experienced at least one transition in the last three months and 1 in 10 patients in the last three days of life. Compared to Spain, Italy and Belgium, patients in the Netherlands were transferred less often at the end-of-life, which might be due to the importance of primary palliative care in this latter country [3]. Our results regarding burdensome transitions were however lower compared to findings in Medicare beneficiaries, where almost 12% had 3 or more hospitalisations in the last 90 days of life and where the percentage of decedents who were transferred in the last three days before death exceeded 14% [10].

In the study by Abarshi et al. [6], 48% of the transitions occurred from home to hospital. The fact that no transitions occurred from hospices and palliative care settings to hospital in their study might be again due to the Dutch system of care with specialist physician-run nursing homes and well equipped hospices and palliative care settings. Hollander and Chappell [34] have found that elderly decedents living at home were transferred more frequently to hospital than patients in long-term care when comparing patients in the same care category level, thereby incurring higher health care costs. The same scenario has been observed by Kaspers et al. [4] comparing transfers at the end-of-life in older people 2000 and 2010 in the Netherlands, as well as in the Belgian study by Gielen et al. [8].

### Strengths and Limitations

Administrative data are very helpful in examining individual patterns and trajectories of health care utilisation as they allow for detailed, population-based analyses over time. They are less susceptible to self-reporting and information biases. However, analyses were conducted retrospectively. In actual practice, the moment when death occurs is unknown. As the study refers to health care utilisation in the last 6 months of life and not to the health care utilisation incurred because of the terminal illness, therefore, the health care utilisation might not be related to the final cause of death.

We might underestimate those dying at a nursing home in the sense that if reimbursing for hospital and nursing homes were present at the time of death, the patient was considered to have died in a hospital. Similarly, hospice use cannot be identified by means of the present claims data. This may have led to a misclassification. However, the proportion of people in a hospice in Switzerland is negligible small. Further, an estimated percentage of 3% of the invoices are not sent to the health insurance, mainly because they are paid directly by the patients. These data could not be included in the analyses. Accident costs were included in the analyses, but as 5% of our sample (mainly persons aged 64 and younger) did not have accident covered by the Helsana Group we might underestimate the number of transitions in decedents aged 64 and younger by around 1.8%. Moreover, in rare cases, two hospitalisations lying close together may have been assessed as one, which may also lead to an underestimation of the number of transitions.

Patients' wishes or treatment focus could not be included which could have further explained transfers between health care settings [6,7]. Therefore, no statement on the quality of care can be made on the basis of the present study findings [35]. Further factors presumably influencing the health care utilisation and transitions (like living arrangement) [36] could not be taken into account as we were lacking this information. Due to the lack of clinical diagnosis chronic conditions were based on medical expenditures (PCGs).

### Implications

The present findings may suggest that not enough persons' preference of dying at home could be complied with. Therefore, timely communication of the preferences, expectations and goals of care at the end-of-life are needed. The Centres for Medicare & Medicaid Services has recently proposed payment for advance care planning conversations between Medicare beneficiaries and physicians or other health care professionals covering, for example, information about the goals of care [37]. In Switzerland too, physicians, ideally primary care physicians, should be rewarded for deliberating with patients their preferences of end-of-life care.

Palliative care services may also help people die at their preferred place of death [14]. Furthermore, international studies have shown that palliative care has the potential to lower heath care costs through a decrease in hospitalisation rates and acute care [11,38,39]. The number of hospital admissions from nursing home could be decreased and the number of people dying at the nursing home instead of the hospital could be increased if enough resources allowed for frequent general physician and nursing visits, as was shown for the UK care home setting [40]. Moreover, home-based end-of-life nursing service has shown to raise the number of home deaths and to lower hospital admissions in England [41]. In almost half of the Belgian patients with life-limiting diseases eligible for palliative care, specialist palliative care wasn't initiated because physicians deemed regular caregiver to be sufficiently skilled [42]. According to their findings, Abel et al. [43] concluded that one third of patients who died in a hospital could have been looked after at home if excellent end-of-life services were available.

Hospitals play an important role in the complex of themes of end-of-life. While some hospitalisations are entirely indicated and appropriate, others (especially burdensome, such as multiple hospitalisations in the last months before death) might have been avoidable [10]. However, the appropriateness of the health care utilisation like hospitalisations was beyond the scope of our study. But monitoring health care utilisation and transitions may reveal regions with potential challenges concerning the coordination of care at the end-of-life. Regional variations in the number of transitions, as has been shown in the present study, imply that either patients have been hospitalised for no clear reason or patients were deprived of medical interventions needed. However, regional factors are modifiable to better meet the patients' preferences and to ensure that the variations reflect the patients' preferences rather than regional differences.

In Switzerland, palliative care is not yet available for all patients in need and there are considerable regional variations. Some regions have a broader supply of palliative care centres, including mobile palliative care teams (i.e. the Western/French speaking part of Switzerland), whereas other regions (i.e. the Central part of Switzerland) hardly have any offering. According to the National Palliative Care Strategy 2013–2015, as little as four out of 26 cantons have hospices available [20]. Furthermore, the financing of palliative care is neither uniformly nor conclusively regulated in Switzerland. And no national regulation exists in terms of hospice funding. Although, the present results of the health care utilisation and the place of death show a need for higher resource allocation. Further research is needed, including patients' preferences, to be able to provide the best possible support for dying patients.

## Conclusions

Health care utilisation was high in the last six months of life and a considerable number of decedents were being transferred at the end-of-life. Timely discussion about the patients' preferences and advance care planning might prevent patients from numerous and particularly from burdensome transitions at the end-of-life.
